# Antibody Mediated Diversification of Primary and Secondary Immune Responses

**DOI:** 10.64898/2025.12.15.694384

**Published:** 2025-12-17

**Authors:** Dennis Schaefer-Babajew, Laurine Binet, Gabriela S. Silva Santos, Chiara Ruprecht, Lachlan P. Deimel, Mohamed A. ElTanbouly, Dounia Gharrassi, Clara Uhe, Kai-Hui Yao, Brianna Hernandez, Parul Agrawal, Anna Gazumyan, Leonidas Stamatatos, Harald Hartweger, Michel C. Nussenzweig

**Affiliations:** 1 Laboratory of Molecular Immunology, Rockefeller University; New York, NY, USA.; 2 Fred Hutchinson Cancer Center, Seattle, WA, USA; 3 Howard Hughes Medical Institute, The Rockefeller University; New York, NY, USA.

## Abstract

Humoral immune responses are characterized by increasing antibody affinity and diversity over time. Increased affinity is mediated by a combination of immunoglobulin gene somatic mutation and iterative cycles of selection in germinal centers. Less is understood about how diversity increases in parallel with affinity. Here we examine the role of antibody feedback in diversifying immune responses in mice that produce B cells that are incapable of secreting antibodies. To this end, we produced two strains of mice, one that expresses only membrane and secreted forms of IgM, and a second that produces only the membrane bound form of IgM. Analysis of primary and secondary immune responses show that antibody feedback significantly diversifies both primary and secondary immune responses even when antibodies are present at levels that are 10–30 fold lower than physiologic. The data have significant implication for sequential vaccination approaches aimed at shepherding immunity to produce broadly neutralizing antibodies to highly diversified pathogens such as HIV-1 and Influenza.

## Summary

Humoral immune responses diversify over time but whether secreted antibodies influence this process is unknown. Using antibody secretion-deficient mice this study shows a profound impact of secreted antibodies on the development of B cell diversity after vaccination.

## Introduction

Both the affinity and diversity of antibodies elicited by immunization or infection increase over time by a process that involves iterative cycles of selection, clonal expansion and somatic mutation in germinal centers (GCs) (Bannard and Cyster, 2017; Victora and Nussenzweig, 2022).

These two apparently discordant features of humoral immune responses, increasing affinity and diversity, can be accounted for by dynamic changes in selection over time and by the existence of two cellular compartments, plasma cells and memory B cells respectively. Plasma cells develop by a mechanism that ensures increasing circulating antibody affinity (Zotos and Tarlinton, 2012; Nutt et al., 2015; Tas et al., 2016; Kräutler et al., 2017; Ise and Kurosaki, 2020; ElTanbouly et al., 2024; MacLean et al., 2025). Memory B cells are selected by a separate mechanism that features diversity over affinity (Suan, Sundling and Brink, 2017; Wang et al., 2017; Viant et al., 2020; Inoue et al., 2021; Inoue, Shinnakasu and Kurosaki, 2022).

Antibody diversification becomes increasingly important during secondary or booster immune responses, when only small numbers of high affinity antigen binding cells can be detected in GCs (Dal Porto et al., 2002; Eckl-Dorna et al., 2019; Mesin et al., 2019; Malladi et al., 2025). Given the strong selection pressure imposed by iterative cycles of mutation and cell division in the GC the evolution away from high affinity is counterintuitive. However, diversification may be an important evolutionary adaptation to deal with rapidly changing pathogens such as Coronaviruses and Influenza virus (Heyman, 2000; Cyster and Wilson, 2024).

Several mechanisms have been suggested to account for the increase in diversity and loss of detectable antigen binding in late primary and secondary GCs. These include: 1. Continual recruitment of additional lower affinity cells during the reaction (De Carvalho et al., 2023; Hägglöf et al., 2023); 2. Increasing T cell fitness that lowers the threshold of B cell selection (Woodruff et al., 2018; Merkenschlager et al., 2023); 3. Antibody mediated antigen masking or enhancement (Heyman, 2000; Cyster and Wilson, 2024).

The effects of antibody feedback on immune responses have been studied since 1909 when they were first described by Theobald Smith in experiments on Diphtheria toxin (Smith, 1909). Recent data shows that masking appears to be mediated by high affinity antibodies and enhancement by low concentrations or low affinity antibodies that may be present even before immunization or in the early stages of the immune response (Heyman, 2000; Cyster and Wilson, 2024). But because all experiments to date, including Smith’s, have been performed in organisms that have at least some level of circulating antibody, precisely how masking and enhancement contribute to the evolution of humoral immunity and GC dynamics remains to be determined. Understanding these phenomena is increasingly important in developing strategies for sequential vaccination for difficult pathogens such as malaria parasites, HIV-1 and Influenza virus where antibody mediated masking can interfere with the evolution of immunity in response to serial vaccine boosters.

Here we examine the role of antibody feedback in regulating GC responses in mice that have an intact polyclonal B cell compartment but do not secrete antibodies.

## Results

### Antibody secretion deficient mice

**Figure 1.**
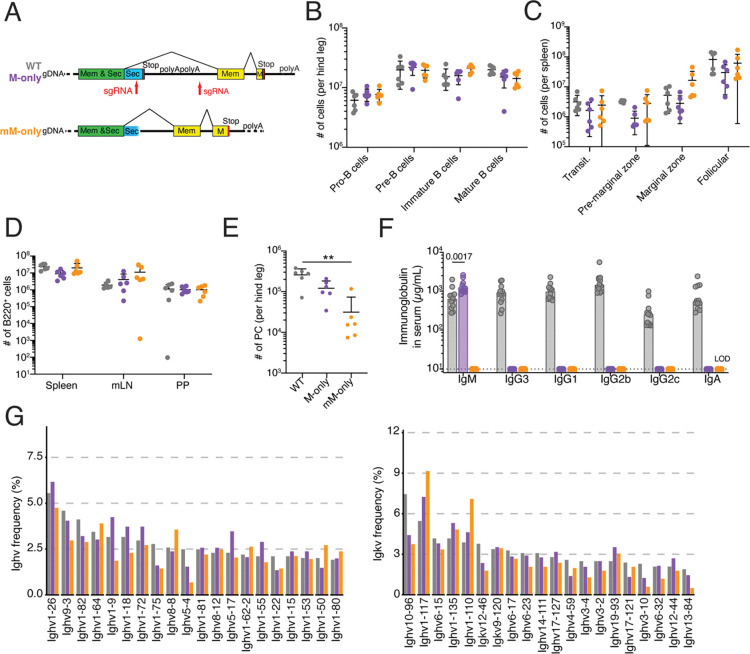
B cell development in M-only and mM-only mice. (**A**) Schematic shows structure of *Ighm* exon 4 in M-only and mM-only mice. (**B**) Number of pro-, pre-, immature and mature B cells in the bone marrow by flow cytometry. Gating as in Supplementary Fig. 1A. (**C**) Number of the splenic B cell subsets by flow cytometry. Gating on B220^+^ cells as in Supplementary Fig. 1B. (**D**) Number of live B cells in spleen, mesenteric lymph node (mLN) and Peyer’s Patches (PP) by flow cytometry. Gating on B220^+^ cells as in Supplementary Fig. 1B–D. (**E**) Number of plasma cells in the bone marrow of one leg by flow cytometry. (**F**) ELISA quantification of IgM, IgG3, IgG1, IgG2b, IgG2c and IgA in the serum of WT, M-only and mM-only mice. (**G**) Ighv (left panel) and Igkv (right panel) gene usage in follicular B cells. Data was pooled from two independent experiments. Each dot or circle represents a mouse. Statistical analysis was by non-parametric one-way ANOVA. All experiments were performed at least twice. **p <0.01. Bars indicate mean and standard deviation.

To examine how secreted antibodies impact the development of immune responses we engineered the IgH locus to produce two strains of mice: a control strain that expresses membrane and secreted forms of IgM (M-only) but no other isotypes; and a second strain whose B cells express only the membrane-bound form of IgM (mM-only) (Figure 1A). To this end, the C57BL/6J Ig heavy chain locus was modified using CRISPR-Cas9 to delete a 150 kb region 5’ of *Ighd* exon 1 to the 3’ UTR of *Igha*, leaving only the IgM locus intact (M-only mice, see methods for further details). M-only mice were subsequently engineered to produce the mM-only mice by removing the stop codon of the secreted splice form and its intronic polyadenylation signal in *Ighm* exon 4 (Danner and Leder, 1985; Nussenzweig et al., 1987; Boes et al., 1998; Ehrenstein et al., 1998; Waisman et al., 2007). The later abrogates production of the secreted form by preventing its termination thereby enforcing splicing to produce the membrane-bound isoform of IgM.

Consistent with the observation that membrane bound IgM regulates B cell development in the bone marrow (Nussenzweig et al., 1987, 1988; Kitamura et al., 1991), M-only and mM-only mice showed generally normal numbers of pro-B, pre-B, immature and mature B cells in the bone marrow (Figure 1B, Supp.Fig.1B–I). Moreover, the numbers of transitional, or mature B2 cells in spleen were similar. As reported by others, mice lacking secreted Igs, show small increases in splenic B1 and marginal zone B cells in the mesenteric lymph nodes but not in Peyer’s patches (Figure 1C,D Supp.Fig.1E–F). There were few if any plasma cells in the bone marrow of mM-only mice and a decrease that did not reach statistical significance in M-only mice (Figure 1E, Supp.Fig.1A,C,D). Serum ELISAs performed under steady state conditions showed slightly increased IgM but no other isotypes in M-only mice and no detectable antibody in mM-only mice (Figure 1F, Supp.Fig.1A, P=0.0017). Despite the absence of other isotypes, single-cell Ig mRNA sequencing showed that the Ighv, Igkv and Iglv usage by follicular B cells was similar in wild type (WT), M-only and mM-only mice (Figure 1G, Supp.Fig.1H). In summary, B cell development appears largely normal, but in the absence of antibody secretion there are no serum antibodies and few if any plasma cells in mM-only mice.

### Primary immune responses

**Figure 2.**
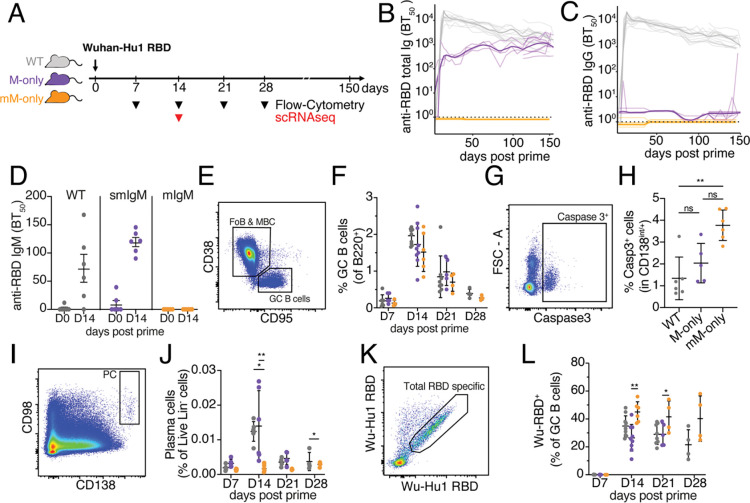
Primary vaccine responses. (**A**) Diagram of the experimental protocol. (**B**) ELISA quantification of anti-RBD antibodies in the serum measured every 3 to 5 days between D0 and D150. (**C**) As in (B) for anti-IgG anti-RBD antibodies in the serum. Thin lines (in B and C) represent single mice, bold lines represent group mean. (**D**) Serum anti-RBD IgM ELISA 0 or 14 after immunization. (**E**) Flow cytometric gating for CD38^−^CD95^+^ GC cells. Pre-gated on B220^+^ cells in draining lymph nodes (dLN). (**F**) Quantification of GC B cells from (E) at the indicated time points after immunization. Each dot represents one mouse. (**G**) Flow cytometry gating for Caspase3^+^ cells among CD138^int/+^ live lin^−^ cells. (**H**) Quantification of (G) among CD138^int/+^ live lin- cells in draining LN at 14 days after immunisaiton. (**I**) Flow cytometric gating for CD98^+^ CD138^+^ PC pre-gated on live lin^−^ cells in the draining LN. (**J**) Quantification of (I) at the indicated times after immunization. (**K**) Flow cytometric gating of RBD^+^ cells among GC B cells in the draining LNs. Pre-gated for GC as in (E). (**L**) Quantification of (K) at the indicatd time points after immunization. Each dot represents one mouse. Statistical analysis using non-parametric one-way ANOVA. All experiments were performed at least twice. Bars in (D, F, H, J and L) indicate mean ± standard deviation. **p <0.01, *p<0.05.

To determine how antibodies impact the development of primary B cell immune responses we immunized mice with the Wuhan-Hu1 SARS-Cov2 receptor binding domain (RBD) (Figure 2A). Antigen-specific antibodies were measured by ELISA (Figures 2B–D). As expected, M-only mice were limited to the IgM isotype, and mM-only mice produced no serum antibodies in response to the immunization (Figures 2B–D). The kinetics of the serological response in M-only mice was similar to WT (Figure 2D), but the total amount of antibody was lower when considering all isotypes (Figure 2B). Notably, whereas the total antibody levels in WT mice decreased over the 150-day period of observation, serum Ig levels were relatively stable and even increased modestly in M-only mice (Figure 2B).

Germinal center responses were analyzed by flow cytometry on days 7, 14, 21 and 28. The absolute numbers of B cells and the relative proportions of germinal center B cells among B220^+^ B cells was similar among WT, M-only and mM-only mice at all time point points analyzed (Figures 2E–F, Supp.Fig.2A). Thus, the contribution of antibodies to GC magnitude and kinetics in the primary response to SARS-CoV2-RBD appears to be limited.

Plasma cell precursors were evident in GCs of all 3 strains, but showed increased caspase expression in mM-only mice and these mice had few detectable mature plasma cells in lymph nodes (LNs) or bone marrow (Figures 2G–J). The data are consistent with the idea that plasma cells that are unable to secrete antibody die by apoptosis (Iwakoshi, Lee and Glimcher, 2003; Todd et al., 2009; Bonaud et al., 2023).

We analyzed the antigen-binding capacity of GC B cells in the primary response by flow cytometry using the Wuhan-Hu1 RBD labeled with 2 different fluorophores (Figure 2K–L). At the peak of the GC response, 14 days after immunization, there were similar proportions of RBD-binding cells in WT and M-only mice (35 % and 26 % respectively), but significantly more in the GCs of mM-only mice (45 % p=0.0021, Fig 2L). Moreover, whereas antigen binding B cells decreased over time in WT and M-only mice they persisted in mM-only GCs (Figure 2L). The data is consistent with the idea that secreted antibodies mask immunodominant epitopes and increase antigen valency, thus curbing the relative competitive advantage of high affinity immunodominant antigen binding cells in the later stages of the primary GC reaction (Heyman, 2000; Andrews et al., 2015; Schaefer-Babajew et al., 2023; Cyster and Wilson, 2024; Yan et al., 2025).

**Figure 3.**
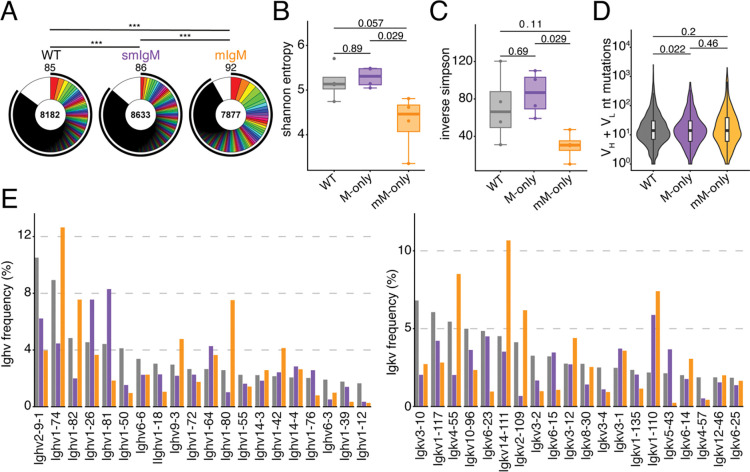
Primary GC antibody sequences. (**A**) Pie charts depicting the distribution of antibody sequences obtained 14 days after prime immunization from GC B cells from 4 mice/genotype. Inner circle numbers indicates the number of sequences analyzed for each genotype. White indicates sequences isolated only once, colored or black pie slices are proportional to the number of clonally related sequences. The outlined black line indicates the percentages of cells in a clonal family. (**B**) Boxplots showing shannon entropy scores for sequences depicted in (A). (**C**) Boxplots depicting inverse Simpson index for sequences shown in (A). (**D**) Violin plots of V gene mutations among sequences show in (A). (**E**) Bar graphs showing Ighv and Igkv gene usage among sequences shown in (A). Sequences used to construct plots in panels (M)-(Q) can be found in Supplementary Table 1. Statistics in (A) indicate chi-squared test with Monte Carlo p-value simulation, p-values were subsequently corrected using the Benjamini-Hochberg (BH) procedure. Statistics in (B)-(D) indicate two-sided Mann-Whitney U test. ****p<0.0001, ***p <0.001,**p <0.01, *p<0.05. Each dot represent a single mouse.

To gain further insights into the effect of antibodies on primary GC responses we examined clonality, diversity and IgV gene usage by single cell VDJ sequencing 14 days after immunization. As expected expanded clones of B cells were found in GCs of all 3 genotypes (Figure 3A). However, the individual clones appeared to be larger and less diverse in the GCs of mM-only mice than in mice that expressed secreted antibodies. Consistent with this observation mM-only GC B cells showed reduced Shannon and Inverse Simpson diversity indices, but similar levels of somatic mutation compared to their counterparts (Figures 3B–D). Moreover, in contrast to the follicular compartment, Ighv gene usage was strongly skewed to *Ighv1–74, ighv1–80, Igkv14–111, Igkv2–109* and *Igkv4–55* in mM-only mice (Figure 3E). In conclusion, secreted antibodies appear to be essential for the development of optimal diversity in primary GC responses to SARS-CoV2 RBD – a mechanism that appears to be significant even at the early stages of the response.

### Secondary Immune Responses

**Figure 4.**
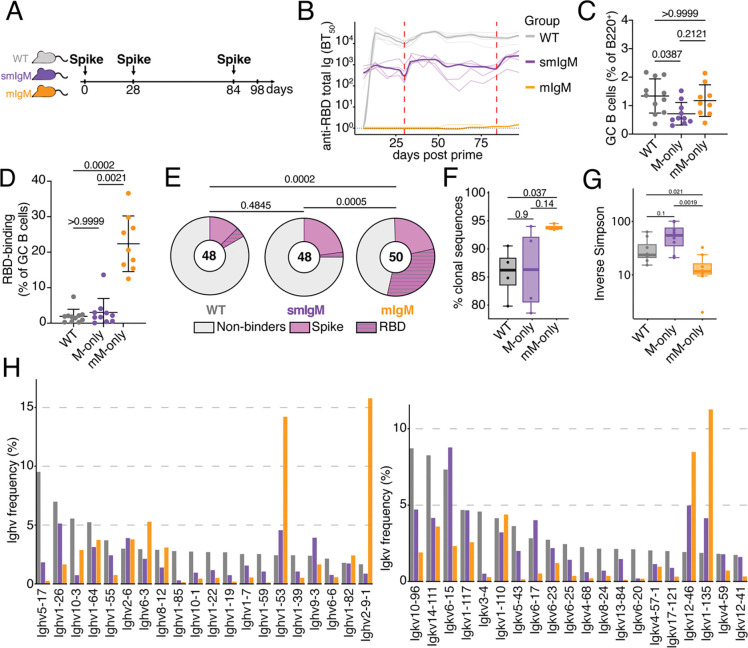
Prime boost vaccination. (**A**) Diagram of the experimental protocol. (**B**) ELISA quantification of total anti-RBD antibodies in the serum measured weekly from D6 to D97 after prime immunization. Thin lines represent single mice, bold line indicates group mean. Dashed red lines indicate booster immunizations. (**C**) Percentage of GC B cells among B220^+^ B cells in the draining LN at D98. Gating as in (Fig. 2E). (**D**) Percentage of RBD-binding GC B cells in the draining LN at D98. Each dot represents one mouse. Gating as in (Fig. 2K). (**E**) Pie charts depicting the distribution of RBD-binding, Spike-binding or non-binding recombinant antibodies cloned from the largest expanded clones of 4 mice/group of GC B cells on D98 from draining LNs. BT_50_ < 10 *μ*g/mLconsidered binders. Inner circle numbers indicate the number of sequences analyzed. (**F**) Boxplots showing the percentage of clonal sequences from GC B cells on Day 98 (**G**) Boxplots depicting the inverse Simpson index for sequences from (F). (**H**) Bar graphs showing Ighv and Igkv gene usage among sequences from (F). Statistics in F and G were determined using the Mann-Whitney U test (two-sided). Bars in (C) and (D) indicate mean ± standard deviation.. All flow cytometry experiments were performed at least twice. Each dot represent a single mouse.

To determine whether antibodies developing during the primary response influence subsequent booster responses, we vaccinated WT, M-only and mM-only mice in accordance with protocols used during human immunization against SARS-CoV2 using full-length stabilized SARS-Cov2 spike protein (Figure 4A).

Serum antibody responses were measured by ELISA at approximately weekly intervals from days 6 to 97 after immunization. As in the primary responses, antibodies were not detectable in mM-only mice (Figure 4B and Supp. Fig. 4A), and M-only mice produced approximately 30-fold less serum antibody compared to WT mice at the peak of the response. Flow cytometry on cells obtained from draining lymph nodes showed that M-only and mM-only mice had overall similar proportions of GC B cells (Figure 4C). Thus, the complete absence of secreted antibodies does not impact the ability to form or sustain secondary GCs.

Antigen binding GC B cells were enumerated using fluorescently labeled Wuhan-hu1 RBD since this is the primary target of the anti-Spike immune response in mice and humans. Notably, the 3 strains differed significantly in the fraction of Wuhan-Hu1 RBD binding B cells (Figure 4D–E). Whereas only 1.9 % and 3 % of the B cells in WT or M-only GCs bound to RBD, 22 % of mM-only GC B cells showed demonstrable binding (p= 0.002, Figure 4D).

To determine the antigen binding specificity of GC B cells that develop after boosting, we cloned and expressed 146 representative antibodies and tested them for binding to Wuhan-Hu1 spike and the isolated RBD by ELISA (Supplementary Table 2). Altogether, only 17 % (wild-type) and 25 % (M-only) of mAbs isolated from secondary GCs exposed to endogenous antibodies showed measurable binding to the immunising antigen, whereas 54 % of the mAbs isolated from mM-only GCs were of sufficiently high affinity to bind by ELISA (Figure 4E and Supp. Fig. 3B). Whereas 32 % of antibodies obtained from mM-only mice bound to the immunodominant RBD, only 4.2 % and 2.1 % of mAbs from WT and M-only GCs did so, respectively (p=0.0002 and 0.0005 respectively, Figure 4E). The fraction of antibodies binding to non-RBD epitopes on the spike was 12.5 %, 23 % and 22 % in WT, M-only and mM-only mice, respectively. To examine the nature of the antibodies produced after booster immunization, we examined the Ig sequences from single cells. Despite similar numbers of somatic mutations (Supp. Fig. 3C), GC B cells obtained from secondary GCs of mM-only mice were more clonal and significantly less diverse than those obtained from M-only mice with notable enrichment in *Ighv1–53* and *Ighv2–91* (Figures 4F–H). The data suggest that antibody feedback inhibits accumulation of GC B cells that are restricted to the initial immunodominant epitope even when those antibodies are restricted to the IgM isotype and when total Ig concentrations are 30-fold lower than physiologic.

**Figure 5.**
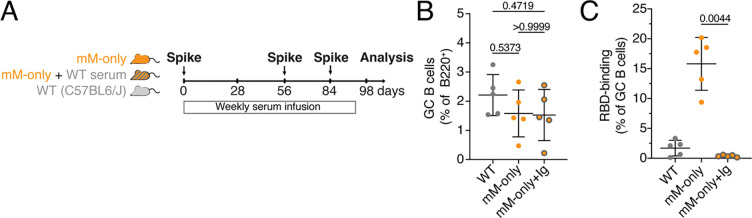
Immune serum rescues the mM-only phenotype. (**A**) Diagram of the experimental protocol. (**B**) Percentage of GC B cells among B220^+^ B cells in the draining LN on day 98. Each dot represents one mouse. Gating as in (Fig. 2E). (**C**) Percentage of RBD-binding GC B cells. Each dot represents one mouse. Gating as in (Fig. 2K). Bars indicate mean ± standard deviation. Statistical analysis was by non-parametric one-way ANOVA. Each dot represent a single mouse.

To determine whether passively transferred antibodies are sufficient to revert the mM-only phenotype we repeated the prime boost experiment with Wuhan-Hu1 spike and transferred contemporaneous serum from WT mice into mM-only recipients (Figure 5A). As determined by ELISA serologic reconstitution was only partial with an approximately 10-fold lower level of specific antibodies in the mM-only recipients compared to their WT counterparts (Supp Fig. 4A). Notably, passive antibody transfer completely reverted the mM-only phenotype resulting in near complete loss of Wuhan-Hu1 RBD binding B cells in the mM-only GCs. We conclude that secreted antibodies make a significant contribution to the specificity and diversity of polyclonal germinal center responses.

**Figure 6.**
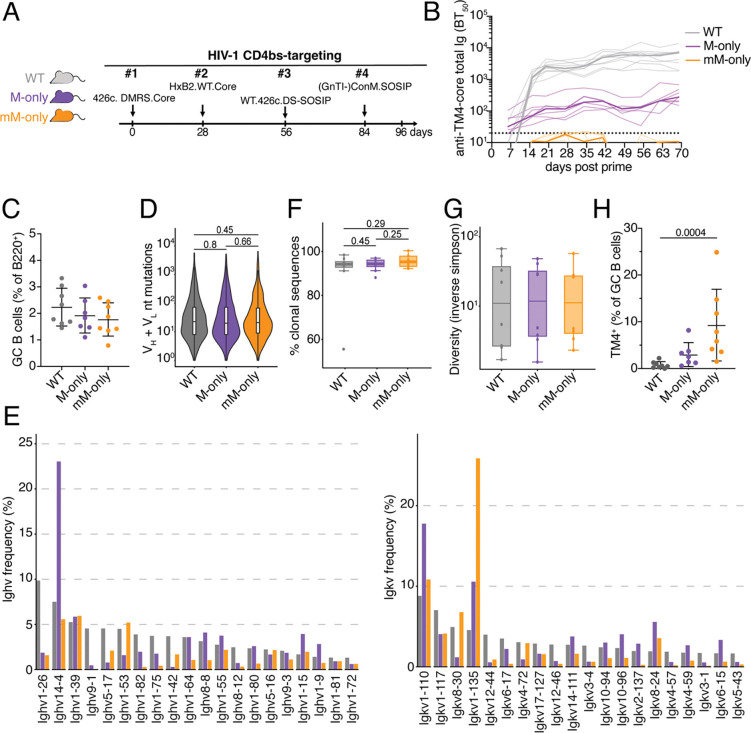
Sequential immunization with an HIV-1 antigen series. (**A**) Diagram of the experimental protocol. (**B**) ELISA quantification of total anti-TM4-core serum antibodies measured weekly from day 6 to 69. Thin lines represent single mice, bold lines represent group mean. (**C**) Percentage of GC B cells among B220^+^ B cells in the draining LN on day 96. Gating as in (Fig. 2E). (**D**) Violin plots showing somatic hypermutation in GC B cells on day 98. (**E**) Bar graphs showing Ighv left panel) and Igkv (right panel) gene usage in GC B cells on day 98. (F) Boxplots depicting the percentage of clonal sequences from GC B cells on Day 98 (**G**) Boxplots depicting Inversed Simpson index of antibody sequences from d98 GC B cells . (**H**) Percentages of TM4 -binding GC B cells. Bars in (C) and (H) indicate mean ± standard deviation. Statistical analysis was by non-parametric one-way ANOVA. Each dot represent a single mouse.

Sequential immunization strategies designed to produce broadly neutralizing antibodies (bNAbs) to HIV-1 and Influenza virus are currently being investigated in animal models and in the clinic. The multi-step vaccine concept being tested involves priming to recruit rare bNAb precursors followed by iterative administration of progressively more mutated antigens to shepherd B cell responses through a series of somatic mutations required to produce bNAbs (Escolano et al., 2016; Burton, 2019; Haynes et al., 2023). This idea has yet to produce necessary protective serologic responses in part because “off target” antibodies dominate the response after booster vaccination (Escolano et al., 2019, 2021). To determine how circulating antibodies might contribute to this effect, we immunized mice with a series of 4 HIV-1 CD4-binding site-targeting immunogens, from which the first one is currently in early clinical testing (NCT05471076; 426c.DMRS.Core (TM4) antigen, Figure 6A) (McGuire et al., 2016). Longitudinal ELISA analysis for antibodies binding to 426c.DMRS.Core showed that titers were present throughout the immunization protocol in WT and M-only but not mM-only mice (Figure 6B).

GC B cell numbers and somatic mutations were equivalent among the 3 mouse strains with median GC B cell frequencies between 1.7 % to 2.2 % of all B cells in the draining lymph node (Figure 6C and Supp. Fig. 6A). Several Igv genes were used preferentially by all 3 groups but at variable frequencies, with only *Igkv1–135* uniquely overrepresented in the mM-only group (Figure 6E and Supp. Fig. 6B). Considering all cells irrespective of HIV-1 antigen-binding, we saw a high degree of clonality in all 3 groups (Figures 6F–G). Notably, there were very few TM4-binding cells in WT and M-only mice but they constituted on average 9 % of all GC B cells in mM-only mice (Figure 6H and Supp. Fig. 5C). Thus, only mM-only mice that are devoid of circulating antibodies show significant levels of antigen-binding GC B cells after a sequential series of HIV-1 vaccine boosters.

## Discussion

GCs are microanatomic compartments specialized for affinity maturation and diversification of humoral immune responses. Affinity maturation is an iterative process involving repetitive cycles of division and mutation in the GC dark zone followed by migration to the light zone where B cells test their newly acquired antigen receptors for binding and capture of antigen deposited in follicular dendritic cells. Selection is mediated by a combination of B cell receptor signaling and T follicular help (Victora and Nussenzweig, 2022; Chen et al., 2023). How GCs diversify antibody responses in the face of strong positive selection is less well understood. Our data indicate that even small amounts of specific antibodies produced during the immune response are sufficient to diversify the GC by masking responses to immunodominant epitopes and by producing immune complexes that lower the affinity thresholds for B cell entry into the GC.

Antibody feedback has been studied primarily by passive antibody transfer in animals and humans (Heyman, 2000; Cyster and Wilson, 2024). These experiments revealed that both IgM and IgG can produce immune complexes and mediate masking through interactions with Fc and complement receptors (Heyman, 2000). Moreover, recent work in antibody knock in mice producing antibodies of differing affinity to a selected epitope showed that the antibody feedback effects were dependent on affinity and that changes in the T cell compartment might also contribute (Barbulescu et al., 2025; Yan et al., 2025). Our experiments extend these findings to primary and secondary immune responses in animals that have an otherwise intact polyclonal B and T cell compartment but are unable to produce secreted antibodies. Our data show that antibody feedback is a major mechanism for diversification of both primary and secondary immune responses.

Diversification by antibody feedback has the potential to enable recall responses to rapidly evolving pathogens. For example, although initial responses to SARS-CoV-2 were focused on highly strain specific epitopes, antibody feedback diversified the response to include more conserved epitopes that offered some protection against newly arising variants. However, our data indicate that these feedback effects pose a very significant barrier to sequential immunization approaches such as those being tested for HIV-1 that aim to focus immunity to a singular broadly neutralizing epitope.

## Methods

### Mice

C57BL/6J mice were purchased from Jackson Laboratories. M-only and mM-only mice were created and maintained at the Rockefeller University with assistance from the Rockefeller University CRISPR and Genome Editing Center and Transgenic and Reproductive Technology Center. Cas9 (IDT) and sgRNA (IDT) ribonucleoprotein complexes targeting the IgH locus were electroporated into C57BL6/J single cell mouse embryos, which were then recovered and incubated overnight at 37 °C before implantation into female foster mother mice. For M-only mice sgRNA with spacer sequences GTAGATCTCTTCCTAAGAGG and TTACTAGGCTCCTCCATATG were used to delete *Ighd*–*Igha*. M-only mice were retargeted and with sgRNAs with spacer sequence CGCCTGTGTCAGACATGATC and GGGTAGGACAAGCAACGCAC to delete the part of *Ighm* exon 4 only found in the secreted IgM splice form, as well as the subsequent stop codon and alternative, intronic polyadenylation site (Danner and Leder, 1985; Boes et al., 1998; Ehrenstein et al., 1998; Waisman et al., 2007). Cutsite adjacent deletions were verified by PCR from tail DNA and Sanger sequencing of PCR products. Sequences were analyzed using Geneious Prime (GraphPad). Mice were bred to homozygosity. Presence or absence of antibody isotypes was verified by flow cytometry and ELISA of serum in homozygous animals from homozygous parents due to maternal transfer of antibodies in utero and through suckling.

Male and female mice aged 6–12 weeks were used. Animals were housed at the Rockefeller University Comparative Bioscience Center and all animal procedures were performed following protocols approved by the Rockefeller University Institutional Animal Care and Use Committee. Animals were housed at an ambient temperature of 22 °C and a humidity of 30–70 % under a 12 h–12 h light–dark cycle with free access to food and water. For experimental endpoints, mice were euthanized using CO_2_, followed by cervical dislocation.

### Immunization

Immunizations were performed by subcutaneous footpad injection of 5 *μ*g of immunogen in 33 % alhydrogel (Invivogen).

### Antibody infusion

Serum was obtained from equally immunized, time-matched C56BL/6J mice and administered to recipients i.v. within 6 h of harvest.

### Flow cytometry

For steady-state experiments, mice aged from 10 to 11 weeks were sacrificed and their spleen, mesenteric LN, Peyer’s patches and bone marrow were isolated and dissociated to obtain single-cell suspensions. Bone marrow from pelvic bone, femur and tibia was isolated by centrifugation at 10,000 g for 15s. Spleen and Peyer’s patches were forced through a 70 *μ*m cell strainer. LNs were collected into 1.5 mL Eppendorf tubes and dissociated using a pestle. Spleen and bone marrow pellets were then incubated in ACK lysis buffer for 15 min on ice, washed and processed together with the LN as described below.

For all experiments, single cell suspensions were incubated in Fc block (BD Biosciences) for 15 min. Cells were then centrifuged at 350 g for 5 min and resuspended and incubated in a solution of PBS diluted fluorescent-Biotin-antigen tetramers for 30 min on ice. Additional labeled antibodies were then added to the cells and samples were incubate for 30 additional minutes on ice. For intracellular staining of Caspase 3, cells were permeabilized using BD Foxp3 permeabilization kit, washed in the supplied permeabilization buffer and stained for 30 min at 4 °C. as per manufacturer’s instructions.

Antigen tetramer staining was performed with a combination of two different fluorescently-labelled streptavidins per antigen. Biotinylated antigens at 5 *μ*g/mL were individually pre-incubated with each streptavidin-fluorophore (all Biolegend) at a 1 *μ*g/mL dilution before staining to allow tetramer formation, then combined and cells resuspend in the mixture and incubated for 30 min on ice, before staining for other surface markers. Samples were acquired on a BD LSRFortessa or BD Symphony A3 or A5. All cell sorting was performed on the BD FACSymphony S6. Data was analyzed using FlowJo 10.10.0.

### Recombinant protein production

All expression vectors for recombinant protein production were confirmed by Sanger (Azenta) or Oxford Nanopore sequencing (Plasmidsaurus). The construct encoding the RBD of SARS-CoV-2 (GenBank MN985325.1; S protein residues 319–539) was previously described (Barnes et al., 2020). All recombinant proteins were produced in Expi293F cells (Gibco, A14527), except 426c.WT.SOSIP which was produced in Expi293F GnTI- cells (Gibco, A39240). Transient transfections of expression plasmids were performed using Expifectamine 293 transfection kit (Gibco, A14525) as previously described (McGuire et al., 2014). In brief, 4–6 days post transfection, culture supernatant was harvested, centrifuged to pellet cells and sterilized by filtration for affinity chromatography. Trimeric Env proteins were purified by passing the supernatant through an agarose-bound *Galanthus nivalis* lectin (GNL) resin (Vector Laboratories) and subsequent size-exclusion chromatography (SEC). A Ni Sepharose 6 Fast Flow resin (Cytiva, 117531803) was used for purification of His-tagged proteins. Native gel electrophoresis identified peak fractions from size-exclusion chromatography. Fractions corresponding to monomeric Envelope trimers, Spike S6P protein trimers or RBD monomers were pooled and stored at −20 °C. Antibodies were purified over a protein G Sepharose 4Fast Flow resin (Cytive 70611805) and buffer exchanged into PBS and stored at −80 °C.

For random biotinylation, proteins were biotinylated using the EZ-Link-Sulfo-NHS-LC-Biotinylation kit according to the manufacturer’s instructions (Thermo Fisher Scientific, 31497). Excess biotin was removed by diafiltration with 100 kDa cutoff. Biotinylated protein was stored at −20 °C or −80 °C.

Avi-tagged TM4 core gp120 or RBD were biotinylated using a BirA reaction according to the manufacturer’s instructions using a 5-fold molar excess of biotin (Avidity, EC6.3.4.15), buffer exchanged to PBS and stored at −80°C.

### ELISA

All ELISAs used Costar 96-well, half area, high binding, polystyrene assay plates (Corning, Cat.# 3960), which were coated with the indicated antigen (TM4, SARS-CoV-2 Wuhan-hu1 RBD or spike protein, all produced in house) or anti-isotype antibody (anti-IgM, Southern Biotech, 1020–01; anti-IgG3, Southern Biotech, 1100–01; anti-IgG1, Southern Biotech, 1070–01; anti-IgG2b, Southern Biotech, 1090–01; anti-IgG2c, Jackson Immunoresearch, 115–005-208; anti-IgA, Southern Biotech, 1040–01) at 2 – 5 *μ*g/mL in PBS over night at 4°C. Plates were blocked with 5 % skimmed milk powder in PBS or 1 % BSA 0.1 mM EDTA 0.05 % Tween20 in PBS for 2 h at room temperature. 6 washes in PBS 0.05 % Tween 20 were performed after every subsequent step. Sera were diluted in PBS at 1:50 to 1:100 top dilution and 1:3 (naïve and after prime) or 1:4 (after boosts) or 1:5 (steady state serum antibody levels) serially diluted for an 8-point curve. Isotype antibody standards (Southern Biotech 5300–01B) were diluted to 10 *μ*g/mL and diluted 1:5 in PBS for an 8-point curve. Secondary antibodies conjugated to horseradish peroxidase were used to detect bound antibodies mouse IgG (1:5000, Jackson ImmunoResearch Cat.# 115–035-071 or Southern Biotech Cat.# 1030–05) or mouse IgM, IgG3, IgG1, IgG2b, or IgA (Southern Biotech Cat.# 5300–05B) or IgG2c (Jackson ImmunoResearch 115–035-208) or total Ig (anti-kappa combined with anti-lambda light chain, Southern Biotech Cat.# 5300–05B). HRP substrate 3,3’,5,5’-tetramethylbenzidine substrate (ThermoFisher, Cat.# 34021) was used for development and the reaction was stopped adding an equal volume of 1 M H_2_SO_4_ (Sigma). Absorbance was read at 450 nm and 570 nm on a FLUOstar Omega (BMG Labtech). For analysis of steady-state antibody concentration in serum, isotype standards were included on every plate and used to fit a sigmoidal 4-parameter logistic regression standard curve in GraphPad Prism 10 which was used to interpolate serum concentrations from dilutions in the exponential phase of the curve. For total anti-antigen responses, sigmoidal 4-parameter logistic regression was fitted to curves of every sample and used to calculate the half maximal binding titer (BT_50_). *μ*g/mL values below detection were set to minimum detection level based on control sera to avoid plotting 0 on logarithmic plots. For samples were signal was too low for a curve fit BT_50_ was set to 10, which is 5x above the highest measured dilution.

### 10x genomics and single-cell libraries

Indicated mice were immunized with either Spike, RBD or HIV proteins. Cell suspensions were prepared as mentioned before from popliteal lymph nodes and samples were indexed using TotalSeqC cell surface antibodies (Biolegend). Live, lineage^−^ B cells (CD38^+^, GC B cells RBD^+^ and GCB RBD^−^), and PC were then isolated flow cytometry and loaded onto the Chromium Controller from 10x Genomics. scRNA-seq libraries were prepared using the Chromium Single Cell 5′ v.2 Reagent Kit (10x Genomics) according to the manufacturer’s protocol. Libraries were loaded onto a Illumina NovaSeq for single-cell gene expression (GEX), VDJ analysis and hashtags (HTO) library at the Rockefeller University Genomics Resource Center. Hashtag indexing from TotalSeqC antibodies was used to demultiplex the sequencing data and generate gene and barcode matrices, respectively.

All single-cell BCR libraries were mapped to the Cell Ranger VDJ GRCm38 reference using Cell Ranger multi v.8.1.0 (10x genomics). Contigs containing less than 50 reads and more than one heavy or light chain were removed. Antibodies heavy and light chains were paired and analyzed using igpipeline v.3.0 (https://github.com/stratust/igpipeline/tree/igpipeline3), previously described (Wang et al., 2023), with the mouse IMGT database as reference (Lefranc, 2011).

For the primary response dataset analyses (Fig. 3), scRNA-seq and Hashtag-oligos unique molecular identifier quantification were performed with Cell Ranger multi v.8.0.1 using the Cell Ranger GEX reference mm10, and analyzed in R with Seurat v.5.1.0 (Hao et al., 2021). Cells were demultiplexed with MULTISeqDemux, and those classified as doublets or with mitochondrial content greater than 10 % and feature count less than 1,000 or greater than 6,500 were excluded. Cell cycle genes were regressed out. Sample batches were then merged, scaled and normalized with SCTransform. B cell (*Cd19, Ms4a1, Cd79a, Tnfrsf13c, Aicda, Lyz2, Cd63*), Follicular (*Cd38, Cd55, Bcl2, Maml2, Notch2, Gpr183, Sell, Ccr6*), and memory B cell (Binet et al., 2024) signatures were assigned using AddModuleScore. Cells with low B cell module score (≤ 25^th^ percentile) or with high memory B cell score (≥ 75^th^ percentile) were excluded. B cells expressing *Bcl6, Aicda, S1pr2* or *Fas* at high levels (≥ 75^th^ percentile) were classified as GC B cells, whereas those with high follicular score (≥ 75^th^ percentile) were classified as follicular B cells.

### Statistical analysis

Details of statistics including tests used, exact values and n numbers are indicated in figure legends and/or main text. Quantification and statistical analyses were performed in R (v4.4.0) with Rstudio server (2024.04.0 Build 735) and/or GraphPad Prism (Version 10.2.3), unless otherwise detailed in this methods section. For all boxplots, the central line denotes the median, the box represents the interquartile range (IQR), lower and upper whiskers denotate the data variability with minimum and maximum data points, respectively, extending 1.5 x IQR from Q1/Q3. Graphs generated using Prism and R were assembled into figures using Adobe Illustrator. Flow cytometry analysis was performed in FlowJo v.10.10.0 software (BD).

## Supplementary Material

Supplement 1

## Data and materials availability

All data needed to evaluate the conclusions in the paper are available in the main text and supplementary materials. Reagents, including mouse strains, are available from H.H. and M.C.N. under a material transfer agreement with The Rockefeller University under reasonable request.
