# Supplementary material for: Antibody Mediated Diversification of Primary and Secondary Immune Responses
Source: bioRxiv. 2025 Dec 17:2025.12.15.694384. Preprint. [Version 1] doi: 10.64898/2025.12.15.694384 (PMC12724515; doi:10.64898/2025.12.15.694384)
Supplement: Supplement 1 [file NIHPP2025.12.15.694384v1-supplement-1.pdf]

bioRxiv preprint doi: https://doi.org/10.64898/2025.12.15.694384; this version posted December 17, 2025. The copyright holder for this preprint (which was not certified by peer review) is the author/funder, who has granted bioRxiv a license to display the preprint in perpetuity. It is made available under aCC-BY 4.0 International license.

Supplementary Figure S1

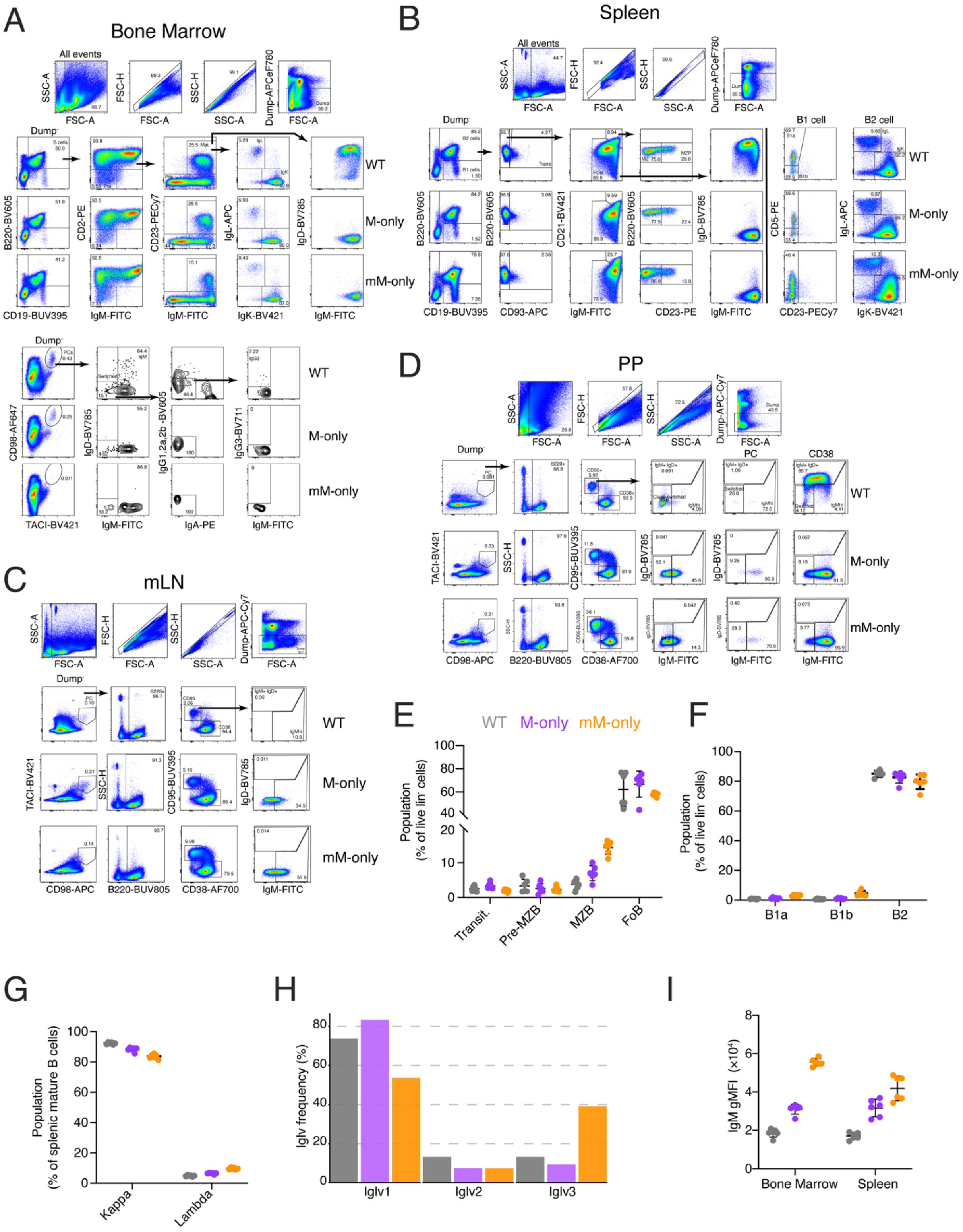

bioRxiv preprint doi: https://doi.org/10.64898/2025.12.15.694384; this version posted December 17, 2025. The copyright holder for this preprint (which was not certified by peer review) is the author/funder, who has granted bioRxiv a license to display the preprint in perpetuity. It is made available under aCC-BY 4.0 International license.

Supplementary Figure 1. Analysis of M-only and mM-only mosue strains. Related to Figure 1. (**A**) Flow cytometric gating strategy in the bone marrow. (**B**) Flow cytometric gating strategy in the spleen. (**C**) Flow cytometric gating strategy in the mesenteric LN (mLN). (**D**) Flow cytometric gating strategy in the Peyer's patches (PP). (**E**) Percentages of splenic, transitional (Transit), pre-marginal zone (Pre-MZB), marginal zone (MZB) , and follicular (FoB) B cells among live lineage^{-} cells. (**F**) Percentages of splenic B1a, B1b amd B2 cells among live lineage^{-} cells. (**G**) Percentages of splenic kappa or lambda light chain bearing mature B cells. (**H**) Bar graphs showing Iglv gene usage in follicular B cells. (*I*) Geometric mean fluorescence intensity (gMFI) of IgM-FITC in the bone marrow mature B cells and splenic follicular B cells. Each dot represent a single mouse. Bars indicate mean ± standard deviation.

bioRxiv preprint doi: https://doi.org/10.64898/2025.12.15.694384; this version posted December 17, 2025. The copyright holder for this preprint (which was not certified by peer review) is the author/funder, who has granted bioRxiv a license to display the preprint in perpetuity. It is made available under aCC-BY 4.0 International license.

Supplementary Figure S2

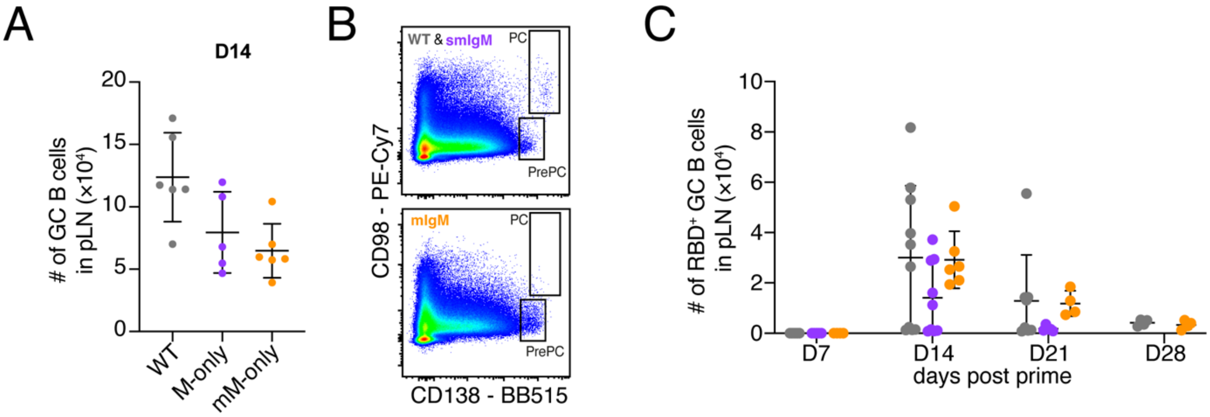

bioRxiv preprint doi: https://doi.org/10.64898/2025.12.15.694384; this version posted December 17, 2025. The copyright holder for this preprint (which was not certified by peer review) is the author/funder, who has granted bioRxiv a license to display the preprint in perpetuity. It is made available under aCC-BY 4.0 International license.

Supplementary Figure 2. Prime immunization with RBD. Related to Figure 2. (**A**) Absolute numbers of GC B cells in draining LN 14 days after RBD immunization. (**B**) Flow cytometric gating strategy for PC. (**C**) Numbers of RBD^+ GC B cells at D7, D14, D21 and D28 after RBD immunization. Each dot represent a single mouse. Bars indicate mean ± standard deviation.

bioRxiv preprint doi: https://doi.org/10.64898/2025.12.15.694384; this version posted December 17, 2025. The copyright holder for this preprint (which was not certified by peer review) is the author/funder, who has granted bioRxiv a license to display the preprint in perpetuity. It is made available under aCC-BY 4.0 International license.

Supplementary Figure S3

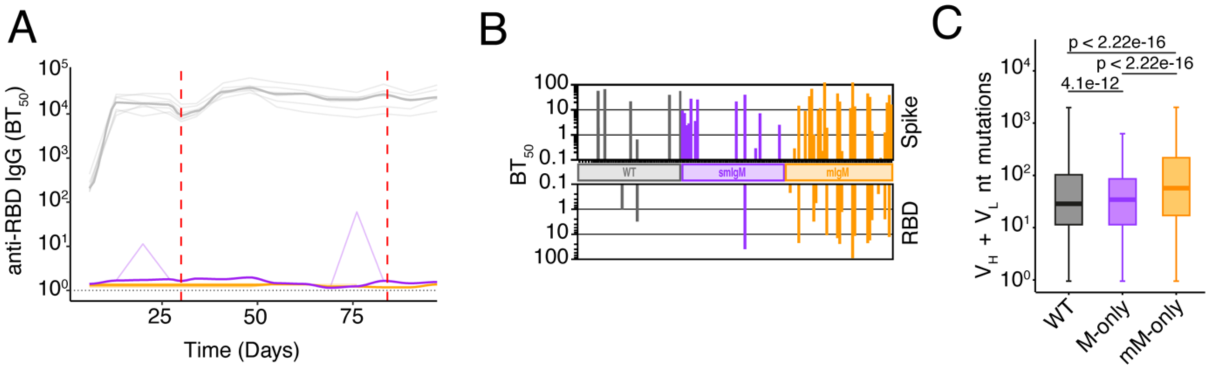

bioRxiv preprint doi: https://doi.org/10.64898/2025.12.15.694384; this version posted December 17, 2025. The copyright holder for this preprint (which was not certified by peer review) is the author/funder, who has granted bioRxiv a license to display the preprint in perpetuity. It is made available under aCC-BY 4.0 International license.

Supplementary Figure 3. Analysis of anti-Spike prime boost responses. Related to Figure 4. (**A**) ELISA quantification of anti-RBD IgG antibodies in the serum of mice from Figure 4 measured weekly from day 6 to day 97. (**B**) Bar graph of recombinant antibodies binding to Spike (top) or RBD (bottom) protein. Each bar represents one antibody. (**C**) Boxplots depicting somatic hypermutations in GC B cells on day 98 from Figure 4 . P-value of two-sided Mann-Whitney U test indicated.

bioRxiv preprint doi: https://doi.org/10.64898/2025.12.15.694384; this version posted December 17, 2025. The copyright holder for this preprint (which was not certified by peer review) is the author/funder, who has granted bioRxiv a license to display the preprint in perpetuity. It is made available under aCC-BY 4.0 International license.

Supplementary Figure S4

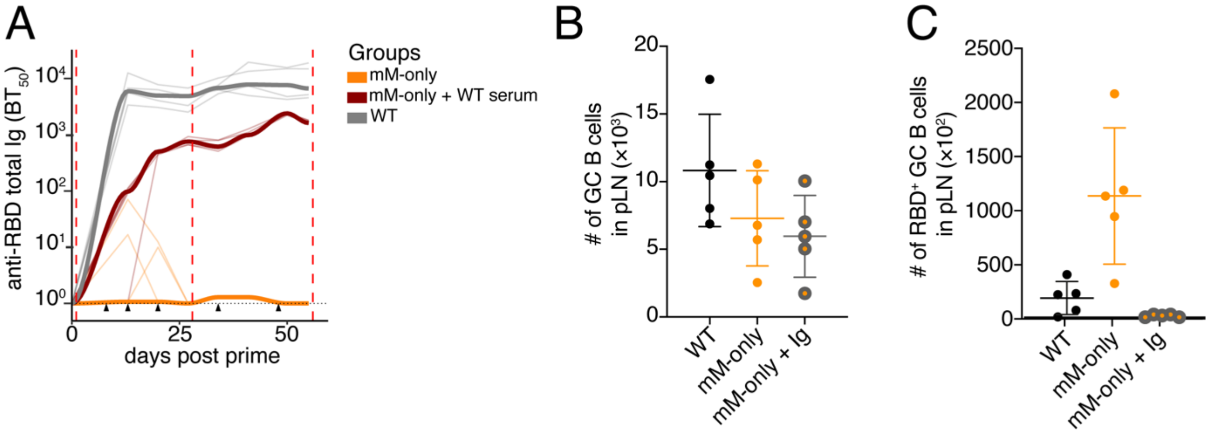

bioRxiv preprint doi: https://doi.org/10.64898/2025.12.15.694384; this version posted December 17, 2025. The copyright holder for this preprint (which was not certified by peer review) is the author/funder, who has granted bioRxiv a license to display the preprint in perpetuity. It is made available under aCC-BY 4.0 International license.

Supplementary Figure 4. Analysis of serum transfer experiment. Related to Figure 5. (**A**) ELISA quantification of anti-RBD total Ig antibodies in the serum measured weekly from day 0 to day 55. Red dashed lines indicate immunizations, black triangles indicate serum transfer. Thin lines indicate individual mice, bold lines indicate mean. (**B**) Number of GC B cells in dLN. (**C**) Number of RBD specific GC B cells in dLN. Each dot represent a single mouse, bars indicate mean ± standard deviation.

bioRxiv preprint doi: https://doi.org/10.64898/2025.12.15.694384; this version posted December 17, 2025. The copyright holder for this preprint (which was not certified by peer review) is the author/funder, who has granted bioRxiv a license to display the preprint in perpetuity. It is made available under aCC-BY 4.0 International license.

Supplementary Figure S5

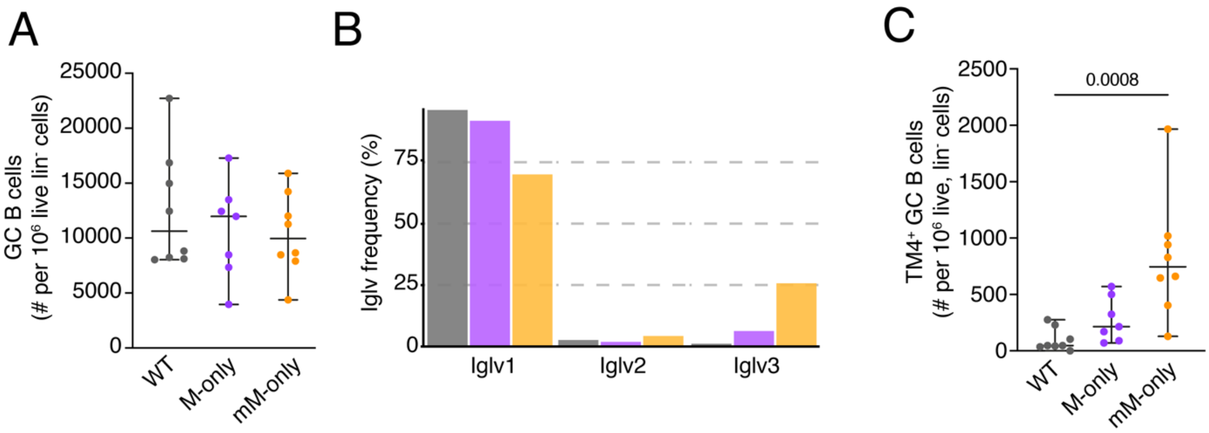

bioRxiv preprint doi: https://doi.org/10.64898/2025.12.15.694384; this version posted December 17, 2025. The copyright holder for this preprint (which was not certified by peer review) is the author/funder, who has granted bioRxiv a license to display the preprint in perpetuity. It is made available under aCC-BY 4.0 International license.

Supplementary Figure 5. Analysis of anti-HIV-1 sequential immunization. Related to Figure 6. (**A**) Number of GC B cells, (**B**) Bar graphs showing Iglv gene usage in GC B cells and (**C**) number of TM4-binding GC B cells in sequentially immunized mice on day 96 in draining LNs. Each dot represent a single mouse, bars indicate mean ± standard deviation.
